# Structural integrity and healing efficiency study of micro-capsule based composite materials via ^1^H NMR relaxometry

**DOI:** 10.1038/s41598-023-39302-3

**Published:** 2023-07-27

**Authors:** S. Orfanidis, M. Kosarli, M. Karagianni, A. S. Paipetis, G. Papavassiliou, M. Fardis

**Affiliations:** 1 Institute of Nanoscience and Nanotechnology, NCSR Demokritos, 15310 Aghia Paraskevi, Attiki, Greece; 2 Department of Materials Science and Engineering, University of Ioannina, 45110 Ioannina, Greece

**Keywords:** Engineering, Materials science

## Abstract

In this work we present a novel approach utilizing nuclear magnetic resonance (NMR) relaxometry to assess the structural stability of microcapsules employed as self-healing agents in advanced aerospace composites both in ambient and harsh environmental conditions. We successfully correlate the amount of the encapsulated self-healing agent with the signal intensity and confirm non-destructively the quantity of the encapsulated self-healing agent mass for the first time in the literature using ^1^H NMR spin–spin relaxation techniques on urea–formaldehyde (UF) microcapsules of different diameters containing an epoxy healing agent. The amount of self-healing agent is shown to increase by reducing the capsule diameter; however, the reduced shell mass renders the capsules more fragile and prone to failure. Most notably, via NMR experiments conducted during thermal cycling simulating flight conditions, we demonstrate that the microcapsule integrity under thermal fatigue varies according to their size. Especially we experimentally verify that the microcapsules with the most sensitive shells are the 147 nm and 133 nm diameter microcapsules, which are the most commonly used in self-healing systems. Finally, we were able to retrieve the same results using a portable NMR spectrometer developed in-house for in situ microcapsule testing, thus demonstrating the potential of NMR relaxometry as a powerful non-destructive evaluation tool for the microcapsule production line.

## Introduction

Self-healing materials are a cutting-edge technology that has piqued the interest of scientists and industry in recent years^1–3^. This is because self-healing materials can repair small to medium-sized lesions and, as a result, restore the material's or structure's disrupted functionalities. There are exogenous and endogenous self-healing abilities, each with their own set of characteristics, benefits, and drawbacks^4^. The intrinsic self-healing approach is based on polymers' inherent ability to recover their original properties via chemical or physical processes such as Diels–Alder or supramolecular reactions ^5–10^. The exogenous self-healing approach, on the other hand, is based on the incorporation of external agents into the material in the form of microtubes (vessels) or microcapsules.

Vascular networks or hollow fibers are used as delivery vessels for the manual or automatic delivery of healing agents to the damaged area. The interconnections of these networks, as well as the properties of the healing agent, have been shown to have a significant impact on the vascular system's healing performance^11–13^. Microcapsule-based composites are potentially the most effective self-healing system that can break the barrier from laboratory-scale studies to aerospace applications, due to the simplicity of design, easy dispersion and minimal reduction in the mechanical properties of the composite structure, as well as the possibility of mass production and quite high healing performance values^14–20^.

In these systems the microcapsules are dispersed in the polymer matrix of the composite. The healing process starts when the healing agent comes in contact with the cross-linking agent, which is dispersed throughout the polymer matrix. Thus, the long-term viability of the system depends on the amount of encapsulated self-healing agent and the quality of microcapsule production. Evidently, accurate and detailed evaluation of both of these factors becomes imperative for quality assurance. Among the techniques used to determine the amount of encapsulated self-healing agent^21–25^, the most prevalent include the determination of encapsulation efficiency (EE) and the High- Performance Liquid Chromatography (HPLC).

In the former method a certain amount of microcapsules is weighed, crushed in a mortar and then washed several times with acetone to dissolve the released core material. The percent microencapsulation efficiency is determined using the ratio of the resulting microcapsules weight to the initial weight of all raw materials. HPLC is used to qualitatively determine the core content of the microcapsules.

Both of the above techniques are destructive by nature; therefore the associated quality tests are only implemented to a limited part of the total microcapsule production. However, in many applications, such as aerospace, shipbuilding and automotive industry, the entire product must be tested to ascertain its performance, necessitating a non-destructive evaluation approach. Non-destructive techniques commonly used in the field of polymer composites include, among others, acoustic emission and infrared thermography, ultrasonic measurements and NMR spectroscopy and relaxometry. In particular, NMR techniques such as ^1^H high and low field NMR, fast field cycling relaxometry and self-diffusion measurements have long been used for the non-destructive evaluation of the structural & dynamic behaviour of polymeric materials.

Field-cycling NMR relaxometry is a well-established method, primarily concerned with the study of the dynamics of a system through the measurement of the T_1_ spin–lattice relaxation^26^. It is a technique that greatly expands the frequency range at which T_1_ can be investigated and thus the spectral frequency of the molecular motions present in a variety of systems in soft matter such as the polymeric compounds considered herein.

Low field studies of soft matter materials are primarily performed in time-domain bench-top NMR spectrometers, see for example^27^ or the mobile single-sided NMR^28^. In the particular case of the epoxy resins they are used primarily with the in-situ characterization of the polymerization and their curing reactions, see for example^29^ where ^1^H CPMG pulse sequences were used to monitor changes in the molecular chain dynamics during the curing process of HTPB and IPDI.

Diffusion NMR is a well-established technique in order to measure the self-diffusion coefficient of spin-carrying molecules in liquids, polymers and other soft-matter materials, see for example^30,31^. It utilizes the NMR technique of spin-echoes, under the influence of an external magnetic field gradient. These NMR measurements are of purely hydrodynamic nature and do not depend on assumptions concerning microscopic characteristics of atomic motion. It is a prerequisite that self-diffusion NMR measurements can only be performed as long as the echo damping of the transversal magnetization due to the migration of the molecules across the inhomogeneous magnetic field is larger than the damping due to the T_2_ spin–spin interactions.

In the present study, we performed ^1^H NMR spin–spin relaxation measurements in static mode on microcapsule batches synthesized in the laboratory at different diameters (64—410.9 μm) in order to investigate the effect of size on the encapsulation efficiency. The microcapsules, consisted of a spherical poly- urea–formaldehyde (UF) shell enclosing Diglycidyl ether of bisphenol-A (DGEBA, Epikote 828 lvel) epoxy resin as the healing agent and were synthesized by in-situ polymerization as described in detail in a previous work^25^. In that work the structural and mechanical properties of the capsules, their thermal stability and healing-agent encapsulation efficiency have been assessed by combined Scanning Electron Microscopy (SEM), Differential Scanning Calorimetry (DSC), Thermogravimetric Analysis (TGA) and ^1^H NMR experiments^25^.

From those experiments a direct correlation between the NMR results and the results of TGA analysis has been experimentally established and it was shown that ^1^H NMR spin–spin relaxation time T_2_ measurements of the self-healing agent can provide in a non-destructive manner accurate and reliable estimates of the encapsulated healing agent content of the capsules.

In the present work we focus on ^1^H NMR spin–spin relaxation measurements and exploit the potential of this technique to investigate the effect of size on capsule tolerance to thermal fatigue and healing agent degradation. In particular:

Signal intensities from different samples of the same batch are compared, to correlate any observed signal loss with the absence of self-healing agent. In this way a quality index can be determined, which quantifies the fragility of the produced microcapsules. Our experiments show that there is an optimal diameter range for which the microcapsules response to mechanical failure is most effective; however, the reduced shell thickness for this size range increases shell vulnerability and the risk of failure during production or transport, necessitating the use of non-destructive evaluation methods.

Thermal stability of the microcapsules is evaluated in-situ with the aid of ^1^H NMR spin–spin relaxation measurements performed during thermal cycling. Temperature variations are one of the most challenging conditions in aerospace structures; therefore, thermal cycling testing is a critical step for ensuring the self-healing agent's integrity in the operating environment. In this work, thermal cycles were performed between − 30 °C and + 60 °C which are the typical test temperatures for aerospace applications. The stability of the capsules under thermal cycling was investigated by measuring room temperature T_2_ values before and after continuous thermal cycles, since changes in T_2_ indicate microcapsule failure and loss of the healing agent. This way, a slight degradation of the healing agent was detected whereas the UF shell remained intact.

Finally, we demonstrate an in-house designed and realized portable NMR spectrometer, able to quantify the healing agent and probe the encapsulation efficiency of the synthesized microcapsules, as was verified experimentally from the observed T_2_ dependence on the microcapsules size.

**Figure 1:**
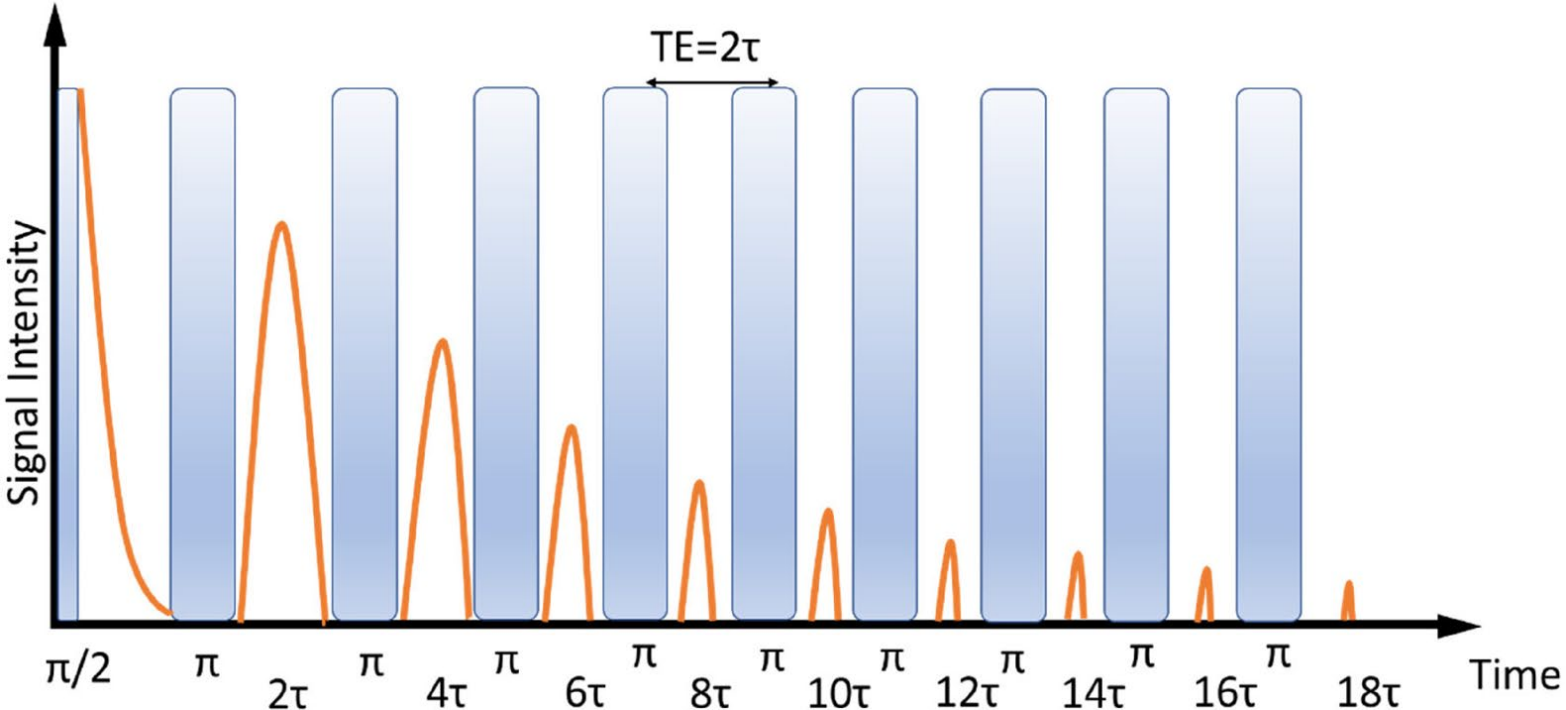
Schematic representation of the CPMG pulse sequence.

All spin–spin relaxation experiments in this work were performed using the Carr-Purcell-Meiboom-Gill (CPMG) pulse sequence, a schematic representation of which is depicted in Fig. 1. In the Carr-Purcell method for the measurement of nuclear magnetic relaxation times T_2_, the spin system is subjected to a π/2, τ, π, 2τ, π, 2τ, … sequence of pulses ((π/2–τ–(π–2τ)_n_) and in the Meiboom-Gill modification, the π/2 pulse is phase shifted by 90^0^ relative to the π pulses to reduce the inaccuracies caused by the inhomogeneities of the rf magnetic field. Essentially, the T_2_ transverse relaxation time can be measured by both the Hahn spin echo and the CPMG pulse train experiments. However, in case of confined liquids and polymers, as is the case of the encapsulated epoxy resins measured herein, internal magnetic fields gradients exist and diffusion effects during the time of experiments cause a rapid decay of the Hahn spin echo yielding lower T_2_ values than the intrinsic spin–spin relaxation time. In a CPMG experiment on the other hand, the effect of internal magnetic field gradients is minimized by the successive application of refocusing π-pulses. Therefore, the later pulse sequence should be used in order to determine the intrinsic T_2_ relaxation time for the encapsulated epoxy resins.

## Results

### Analysis of capsule surface morphology and size distribution with Scanning Electron Microscopy (SEM)

**Figure 2:**
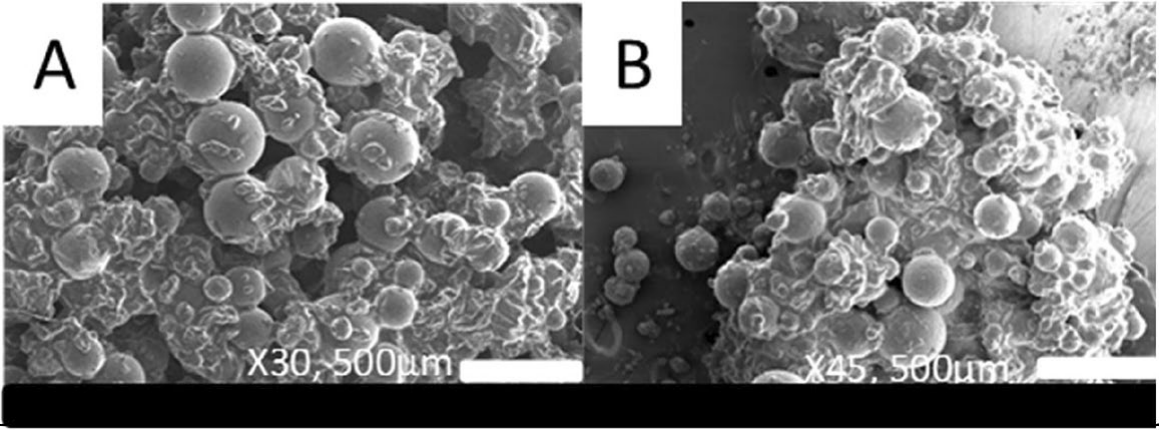
SEM images from microcapsules prepared at agitation rotational speeds of (**A**) 200 rpm and (**B**) 800 rpm.

Poly- urea–formaldehyde (UF) microcapsules containing the epoxy resin Diglycidyl ether of bisphenol-A (DGEBA, Epikote 828 lvel) as a healing agent were synthesized by an in-situ polymerization method described in a previous work^25^. Capsules of different diameters were produced by appropriate adjustment of the agitation speed in the stirring stage of the synthesis process^25^. The morphology and size of the capsules were analyzed by Scanning Electron Microscopy (SEM). Figure 2a,b display SEM images of microcapsules fabricated at agitation rotational speeds of 200 and 800 rpm respectively. SEM investigation revealed that the capsules had a rough exterior surface and a spherical form. By virtue of this particular structure, the mechanical interaction between the capsules and the polymer host matrix is increased, leading to improved stability of the material. According to SEM analysis, the average diameter of microcapsules produced at various agitation rotational speeds varied significantly, with values of 410.9 μm, 306.7 μm, 205.8 μm, 147.2 μm, 133.5 μm 34.5 μm, and 64 μm for capsules produced at 200 rpm, 300 rpm, 400 rpm, 500 rpm, 600 rpm, and 800 rpm respectively. These findings indicate that rotational speed is an important indicator of microcapsule size, with lower agitation speeds producing larger capsules and higher speeds producing smaller capsules.

### Thermogravimetric analysis (TGA)

TGA measurements were performed on all six microcapsules batches in order to evaluate the thermal stability of the synthesized samples. The results are summarized in Table 1. In all investigated samples mass loss was observed to occur in two main steps^25^: the first step was observed in the temperature range 180 °C to 320 °C and was attributed to thermal disintegration of the capsule shell and onset of solvent evaporation, while the second step, observed in the temperature range 320 °C to approximately 485 °C was attributed to homo-nuclear polymerization of the healing agent resin^25^. Most notably, from Table 1it is inferred that the percentage of encapsulated healing agent increases with decreasing capsule diameter indicating increased encapsulation efficiency in the smaller capsules.

**Table 1:** Thermogravimetric analysis results.

Mean diameter (μm)	Tonset (°C)	Mass of shell wall and solvent (%)	Encapsulated healing agent (%)	Remaining mass (%)
410.9 ± 21.9	181	25.9	53.6	20.5
306.7 ± 20.60	210	20.6	58.2	21.2
205.8 ± 26.3	213	12.8	64.7	20.8
147.2 ± 25.4	218	11.7	71.5	16.8
133.5 ± 34.5	223	10.5	69.9	19.4
64 ± 27.6	230	8.6	73.5	17.6

### Solid state nuclear magnetic resonance (NMR)

#### Evaluation of the quantity of the encapsulated self-healing agent

Solid-state NMR relaxometry was used to monitor the successful encapsulation of the healing agent non-destructively. In particular, the observed ^1^H NMR signal intensity was correlated with the amount of encapsulated healing agent. However, the acquired NMR signal is expected to include contributions from hydrogen atoms in both the solid shell wall and the liquid core. Nevertheless, the signal from hydrogen atoms in the solid UF shell has been shown to decrease fast due to the restricted mobility of UF protons and essentially disappears at approximately τ = 60 μs, as shown in Supplementary Figure S2. Therefore, the NMR signal was acquired after a suitable time interval τ = 600 µs, to ensure that the recorded echo signal originates entirely from the ^1^H nuclei of the liquid resin-solvent mixture.

**Figure 3:**
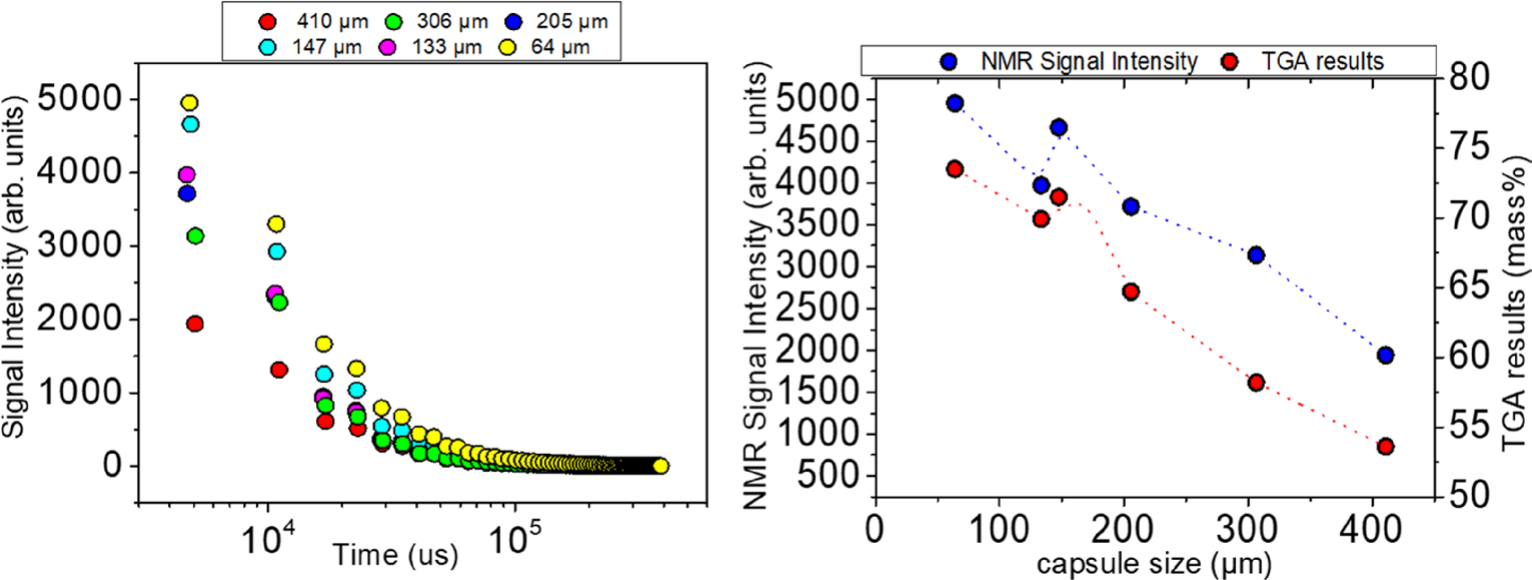
(**A**) Room temperature ^1^H NMR CPMG echo decay curves acquired at 200 MHz from all microcapsules batches. (**B**) Correlation of NMR and TGA measurements.

Figure 3A depicts the CPMG spin echo decays (SEDs) acquired at room temperature from microcapsules samples of different diameters, but of the same total sample mass (7 mg). In the graph of Fig. 3B the NMR signal intensity is plotted as a function of the mean capsule’s diameter. In the same graph the corresponding mass percentage of encapsulated healing agent calculated from TGA measurements is also shown. The NMR signal intensity is observed to gradually decrease with increasing capsule size in both Fig. 3A,B. Since the intensity of the acquired ^1^H NMR signal is proportional to the number of the detected ^1^H spins in the sample, the observed signal decrease can be assigned to a lower liquid content of the larger capsules compared to the smaller ones. Furthermore, from Fig. 3B a direct correlation can be established between the NMR signal intensity and the mass percentage of encapsulated healing agent measured by the TGA.

**Figure 4:**
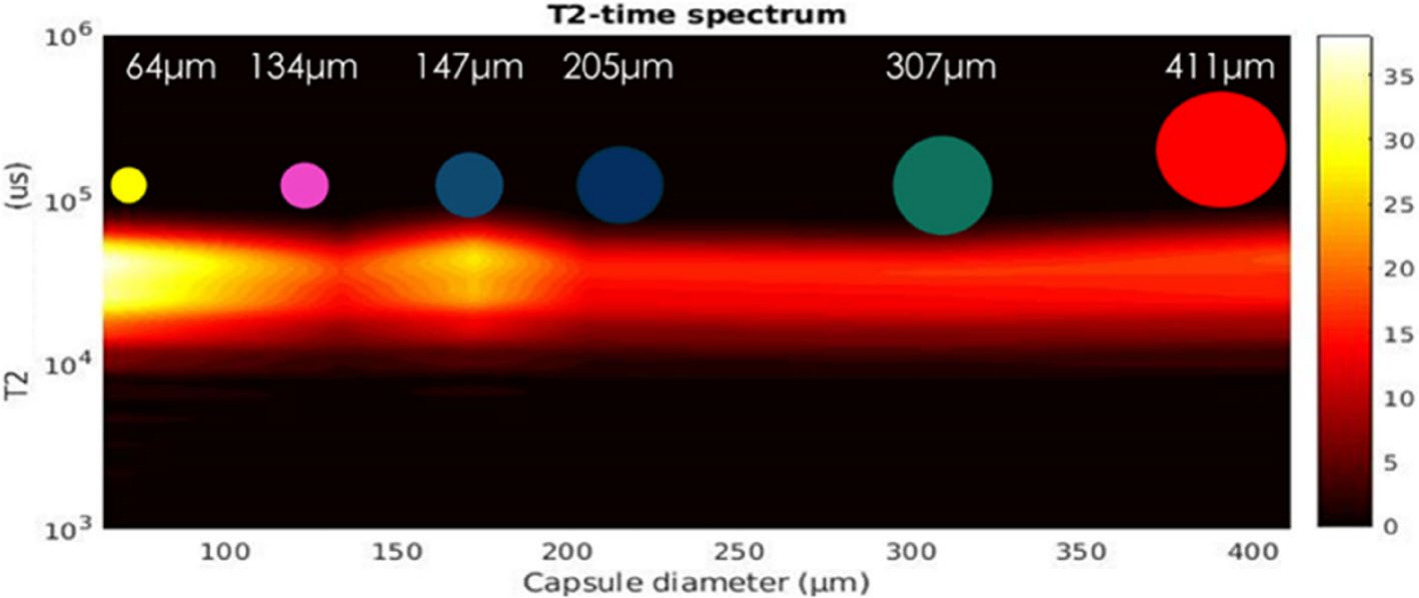
Distribution of the ^1^H spin–spin relaxation time T_2_ as a function of the mean microcapsule diameter. The NMR signal intensity varies according to the color map shown on the right.

The decrease of the NMR signal intensity with increasing capsule size is also mapped in the contour plot of Fig. 4 which illustrates the distribution of the relaxation time T_2_ as a function of the mean capsule diameter. More important, it is observed in Fig. 4 that the measured values of the relaxation time T_2_ are the same regardless of the microcapsule size. Taking into account that T_2_ probes the dynamics of molecules entrapped in the interior of the microcapsules, this is strong indication that the dimensions of the capsule's internal cavity are several orders of magnitude larger than the size of the epoxy resin molecules (probably on the order of micrometers). Indeed, under strong confinement in cavities with dimensions comparable to those of the confined molecules, a reduction of T_2_ with decreasing capsule size would be expected due to the strong interaction of the confined liquid with the internal walls of the cavity.

#### Monitoring the structural integrity of the produced capsules

**Figure 5:**
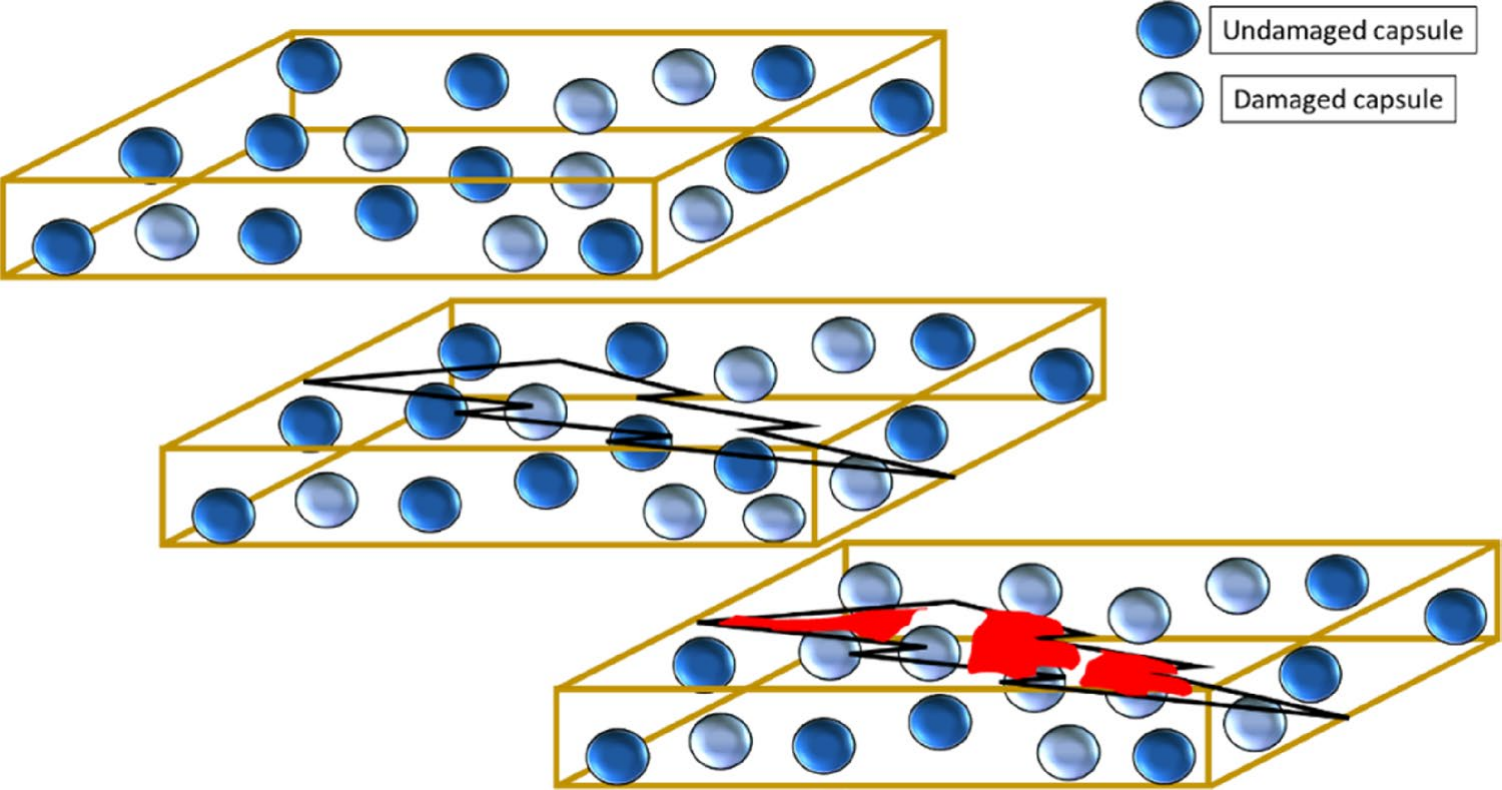
Schematic presentation of the healing process in the presence of ‘pre-damaged’ micro-capsules.

The quality index quantifies the vulnerability of the micro-capsules prior to their introduction into the polymeric matrix and how defective microcapsules can undermine the final structure and healing efficiency, as schematically shown in Fig. 5.

**Figure 6:**
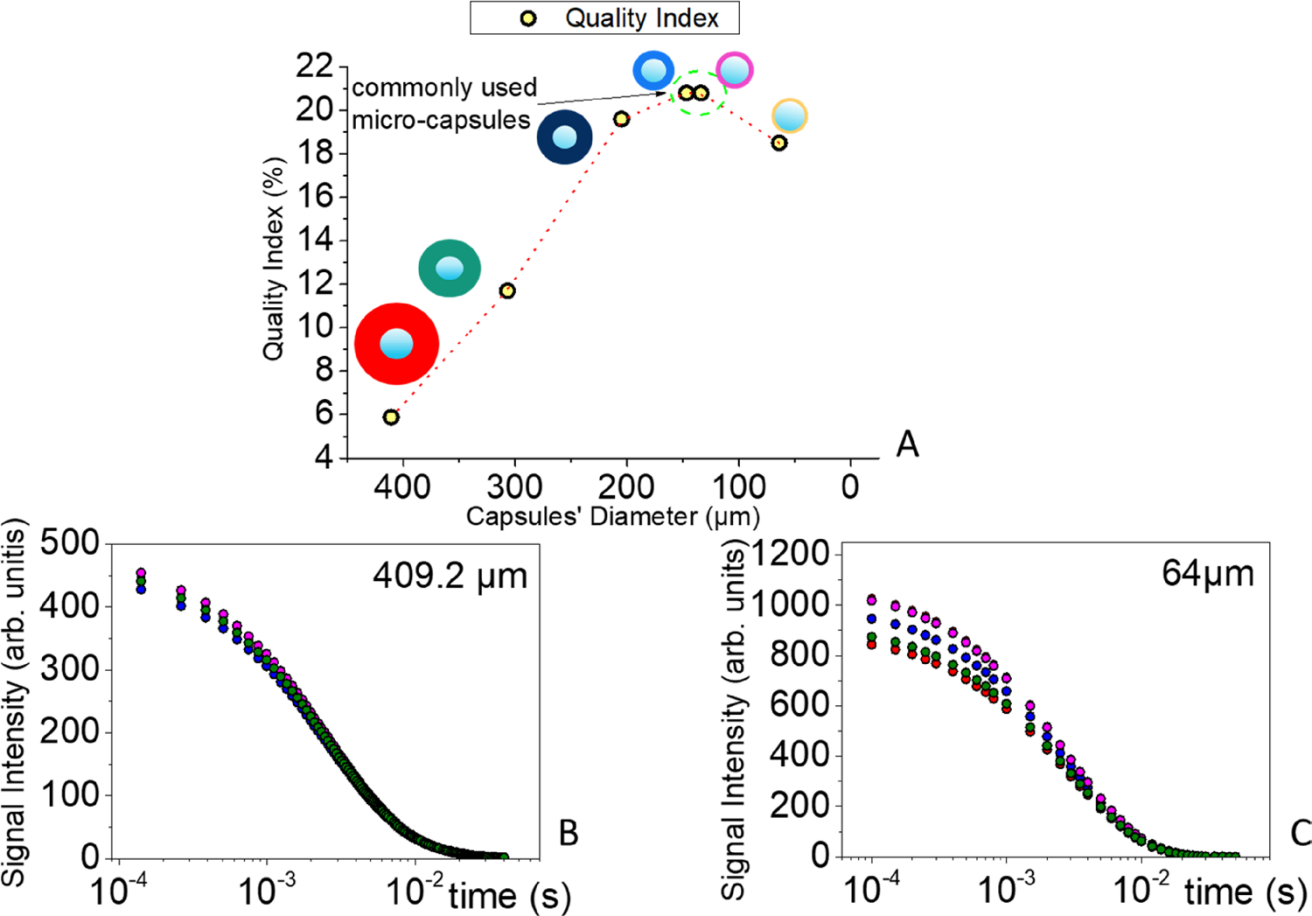
(**A**). Correlation of the quality index to capsules’ shell thickness. (**B**), (**C**). ^1^H NMR CPMG decay curves of micro-capsules samples with a mean diameter of 410.9 μm & 64 μm respectively.

The defective capsules content was evaluated for each capsule batch by comparing the NMR signal intensity from different samples of the same batch. Figure 6B,C display the ^1^H NMR CPMG decay curves for the larger (410.9 μm) and smaller (64 μm) microcapsules respectively.

The corresponding curves for all batches are given in the Supplementary Information (Figure S3). It is clearly observed that the fluctuations in the NMR signal intensity between samples of the same batch increase as the capsule diameter decreases, becoming more prominent for the smaller capsules.

As discussed above, a decrease in the observed signal intensity indicates a reduced mass percentage of the self-healing agent. Therefore, fluctuations in the signal intensity between different samples of capsules of the same diameter, obtained from the same batch, imply the presence of defective capsules (either empty or with a ruptured shell releasing the healing agent).

Figure 6A illustrates the quality index of the microcapsule batch in relation to the capsule external diameter. Taking into account the confirmed hypothesis that shell wall thinning takes place with decreasing capsule size^32^, the observed fluctuations in signal intensity for the smaller capsules may be attributed to increased fragility due to shell thinning. It can be seen in Fig. 6A that the capsules at mean diameters 147.2 μm and 133.5 μm are more likely to fail during microcapsule production or transport; however, when immersed in the polymer matrix, they would easily respond to any mechanical disturbances of the structure. It may be anticipated therefore, that microcapsules of these diameters are the most suitable for aerospace applications.

However, the observed increase in signal intensity fluctuations with decreasing capsules size implies a higher percentage of defective capsules in the smaller capsules batch. This is an issue of great importance, since the presence of even a small percentage of defective microcapsules can reduce the overall healing efficiency of the system and undermine the integrity of the final healed structure. Indeed, defective capsules do not contribute to the healing process and leave unhealed damaged areas that promote the development of larger cracks in the polymer matrix. This is schematically illustrated in Fig. 5, which depicts the evolution of the healing process in the presence of defective (light blue) and non-defective (dark blue) microcapsules initially dispersed in a polymer matrix. As shown, a propagating crack encountering non-defective capsules will cause the rupture of the capsule shell and the release of the healing agent, thus activating the healing process, whereas defective capsules allow crack propagation, thus degrading the healing process^33,34^.

**Figure 7:**
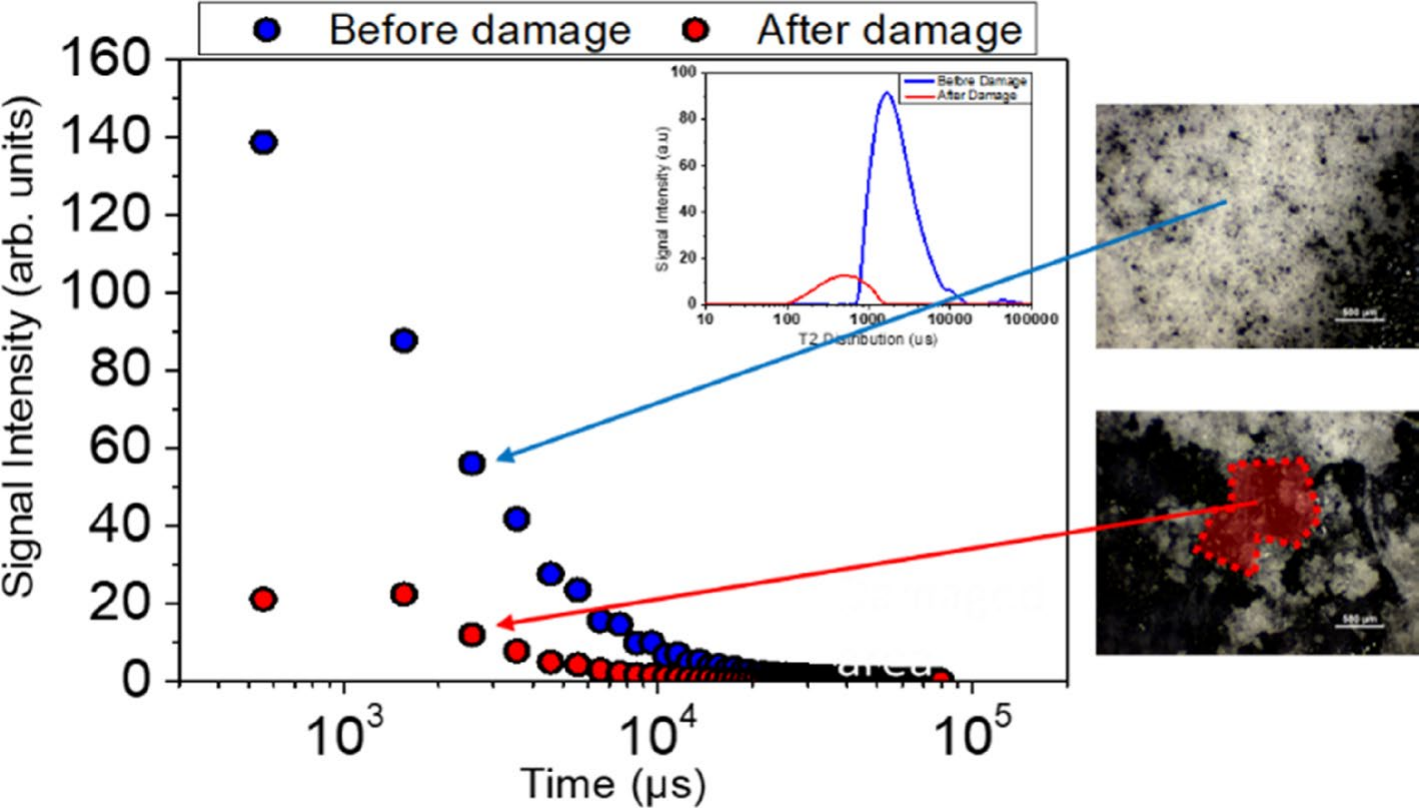
^1^H NMR signal intensity of micro-capsules with mean diameter 410.9 μm before and after creation of the defect. The optical microscope images shown on the right illustrate the damaged area.

To make the correlation between the loss of signal intensity and the absence of self-healing agent more obvious, we created a defect in a sample specimen and compared the optical microscope images and NMR signal intensity plots before and after the fraction. As shown in Fig. 7, in addition to the loss of signal intensity, the damage also affected the characteristic relaxation time T_2_ which is observed to shift to lower values after damage due to solvent evaporation. In the optical microscope images, the undamaged microcapsules appear as bright white spheres, while the damaged area appears darker as the shells were immersed in the self-healing agent.

**Figure 8:**
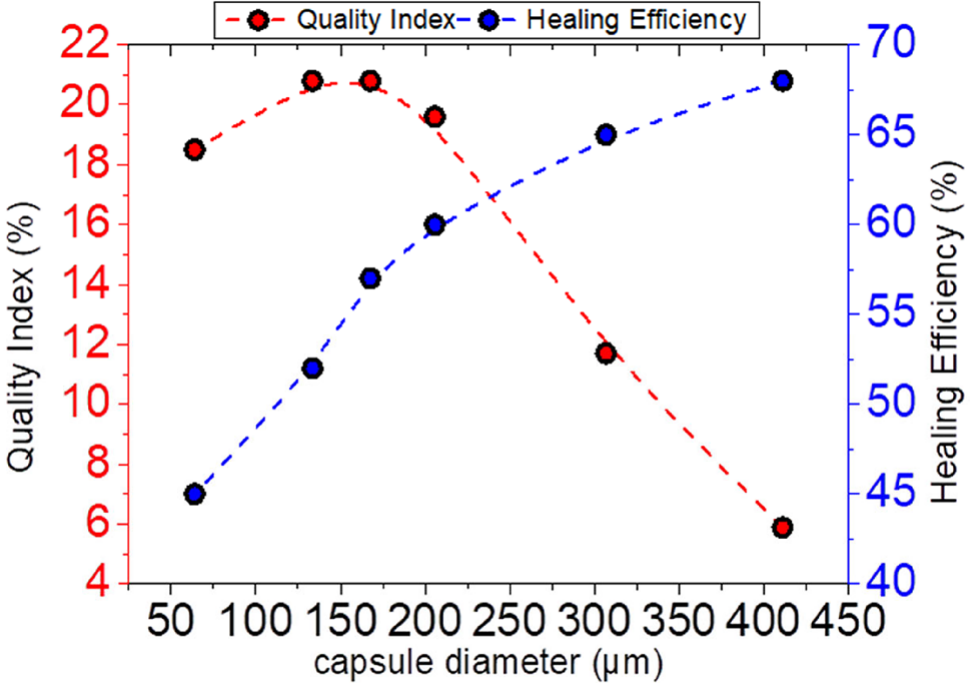
Correlation of the quality index to the self-healing efficiency as a function of capsule diameter.

According to results published in our previous work^25^, the smaller size microcapsules have a high encapsulation rate but low self-healing efficiency performance which was attributed to capsule pull-out and insufficient micro-capsules. The present results demonstrate that the lower self-healing efficiency of the smaller capsules may be also well explained by the higher percentage of defective capsules. This is most clearly seen in Fig. 8 which depicts the correlation between the healing efficiency calculated in our previous work for the various capsule sizes^25^ and the corresponding quality index determined in the present work from the quantitative analysis of the results shown in Fig. 6B,C.

Nevertheless, the above findings confirm that non-destructive quality evaluation of the capsules is required due to the capsule shell's vulnerability.

### Thermal cycling test

The most demanding conditions in aerospace applications are mechanical and thermal fatigue. In order to examine the stability of the shell structure during thermal cycling, we conducted an experiment simulating the thermal fatigue of the materials under actual operating conditions.

Three microcapsule batches with outer diameters of 410.9 μm, 147.2 μm and 64 μm were subjected to three consecutive thermal cycles as described in the Materials and Methods section. In each cycle, the distributions of ^1^H T_2_ values were acquired at -30 °C and + 60 °C. For each batch T_2_ values were also acquired at room temperature (+ 25 °C) before and after the thermal fatigue test.

**Figure 9:**
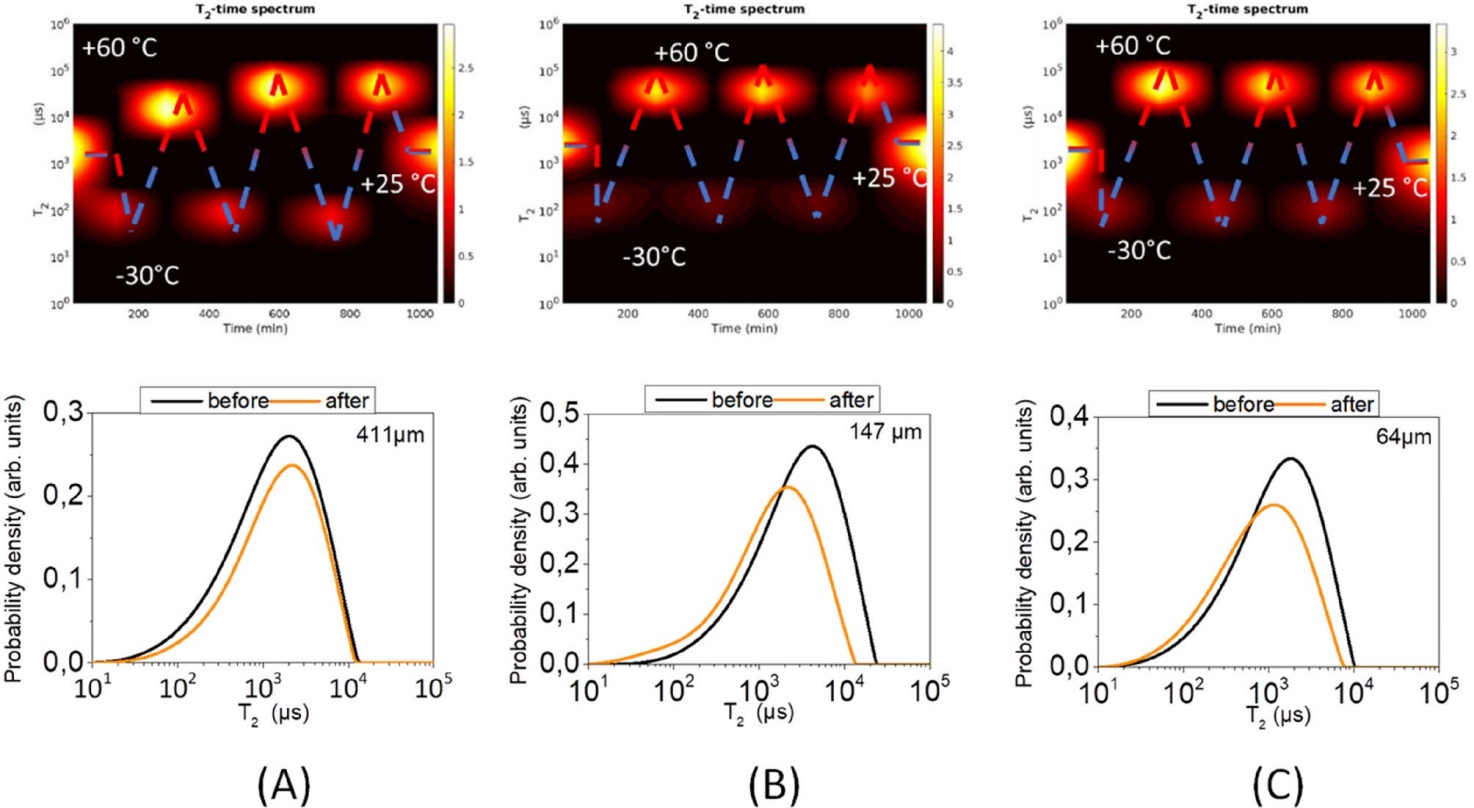
^1^H T_2_ contour plots (upper panel) and distributions (lower panel) throughout a three thermal cycles process for microcapsules of mean outer diameter (**A**) 410.9 μm, (**B**) 147.2 μm and (**C**) 64 μm. Red and blue regions designate T_2_ values at + 60 °C and − 30 °C respectively.

The contour plots in the upper panel of Fig. 9 display the evolution of the T_2_ distribution throughout the thermal cycling process for each of the three batches (Fig. 9A–C). The graphs in the lower panel show the corresponding room temperature T_2_ distributions at the beginning and at the end of the thermal cycling process.

In Fig. 9 an increase in the T_2_ value of the healing agent at + 60 °C is observed for the larger capsules after the first thermal cycle (Fig. 9A), which may be attributed to a self-diffusion motion with increased mobility of the healing agent molecules. No such behavior was detected in the two smaller diameter (142 μm and 64 μm) capsules (Fig. 9B,C respectively) due to the significantly smaller available space of the inner capsule cavity.

Most importantly, it is observed that in the larger capsules (410.9 μm outer diameter) the T_2_ values at room temperature remain the same before and after thermal fatigue. On the other hand, for the 147.2 μm and 64 μm outer diameter capsules a decrease in the room temperature T_2_ value is observed at the end of the thermal cycling process, by 24% and 38% respectively compared to the corresponding T_2_ values at the beginning of the process. This T_2_ shift is primarily attributed to solvent evaporation through some cracks on the capsule surface, rather than to structural collapse of the capsule shell, since in the latter case a dramatic change in the room temperature T_2_ values should be observed after the completion of the thermal cycles.

The decreased tolerance to thermal fatigue of the smaller capsules compared to the larger ones is to be expected on the basis of the results reported in Fig. 6, where it is shown that these batches have the thinnest capsule shell and the highest damage rate.

### Portable solid-state nuclear magnetic resonance spectrometer

It was shown in Fig. 6B,C and in Figure S3 of the Supplementary Information that the intensity of the ^1^H NMR signal from different samples of capsules of the same diameter exhibits fluctuations. These were attributed to the presence of defective microcapsules and were observed to become more pronounced as the size of the microcapsules decreases and the capsule shell becomes thinner.

In order to minimize the chances of damaging the microcapsule production through transport, we developed a portable solid-state NMR spectrometer to be used for in-situ testing.

**Figure 10:**
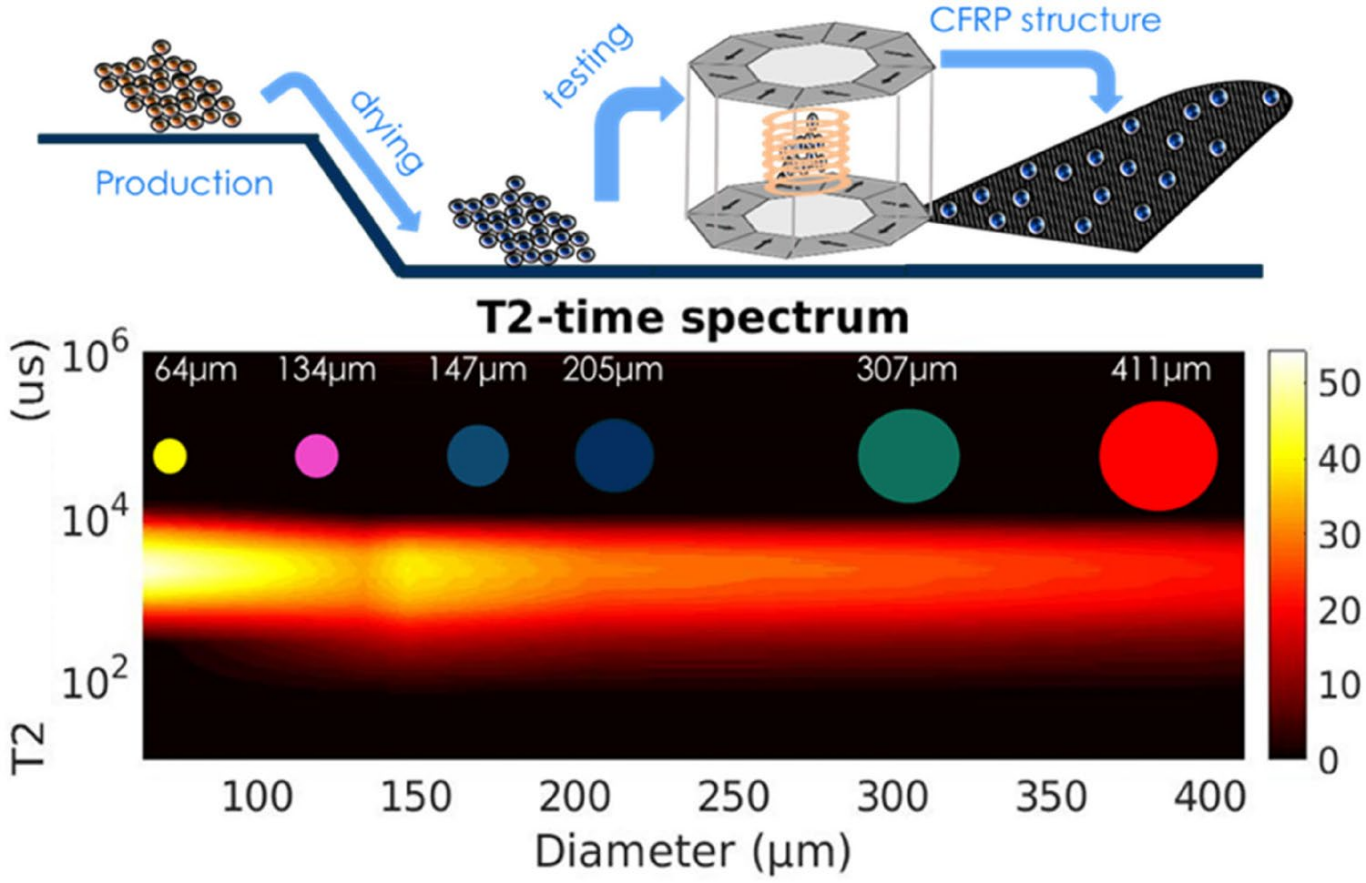
Top: Schematic of the portable NMR spectrometer and its use in a laboratory production line. Bottom: NMR signal intensity of micro-capsules samples of each batch (410.9 μm–64 μm).

To facilitate its transport, the spectrometer is equipped with a small size, low magnetic field (0.29 T) Halbach permanent magnet. The upper panel in Fig. 10 gives a schematic description of the device and its use in a laboratory production line. The fidelity and proper operation of the portable spectrometer were assessed by measuring the ^1^H NMR relaxation time T_2_ of the healing agent in all batches, in the same way as in the high field case. The corresponding distribution of the relaxation time T_2_ as a function of the mean capsule diameter is shown in the bottom panel of Fig. 9. A direct comparison of Figs. 4 and 10 clearly shows that the results acquired with the portable spectrometer are the same as those obtained in the high field measurements. In particular, apart from the shorter T_2_ values obtained with the portable spectrometer due to the lower field of the Halbach magnet^35^ (0.29 T as compared to the 4.7 T in the high field measurements), T_2_ is seen to remain the same for all capsule sizes as was also observed in the high field case. Moreover, by increasing the capsule diameter the signal intensity decreases in the same way as also observed in Fig. 4, therefore confirming the successful operation of the portable spectrometer.

## Discussion

In conclusion, we have successfully evaluated non-destructively the quality of the entire microcapsule production, for the first time in the literature employing solid state NMR relaxometry. As shown in Fig. 6, the variation in the NMR signal intensity across different samples from the same batch is induced by the presence of defective capsule shells, thus giving a “suitability criterion” regarding the applicability of the microcapsules in self-healing applications.

Most important, the microcapsules which appear to be more prone to fractures in the shell are microcapsules with diameters of 147 nm and 133 nm which have also a high quality index approaching 20% as shown in Fig. 6A. These micro-capsules seem to be the most preferable since their structure does not significantly affect the mechanical properties of the final structure as was mentioned in our previous work Kosarli et al.^25^. These facts demonstrate the necessity of a new non-destructive approach, such as solid state NMR, to examine this type of materials and structures and evaluate the effectiveness of the self-healing structure.

To further test the tolerance of the microcapsules shell integrity in flight conditions, we subjected the capsules to continuous thermal cycling since temperature variations constitute a major challenge in the aerospace field. The results were encouraging as Fig. 9 shows that the microcapsules’ shells were intact after the thermal cycles. On the other hand, the encapsulated self-healing agent showed a slight degradation as the spin–spin relaxation time T_2_ was observed to shift to shorter times. Since ${\mathrm{T}}_{2}\propto 1/\upeta$, where *η* is the fluid viscosity, the T_2_ shortening might be due to the separation of the epoxy resin DGEBA and the solvent EPA during the thermal cycles.

Overall, our findings show that NMR is a powerful non-destructive evaluation technique well suited to providing the highest level of quality control required by the aerospace industry.

## Methods

### Materials

The materials and mixing procedure were identical to those described in our previous work. Kosarli et al.^25^. Details on microcapsules synthesis and the encapsulation process are also provided in Section 1.1 and 1.2 of the Supplementary Information. In a nutshell, diglycidyl ether bisphenol-A (DGEBA, Epikote 828) epoxy resin diluted with ethylphenyl acetic acid (EPA at 5% w/w) was added to an aqueous solution of poly (ethylene-alt-maleate-anhydride) copolymer powder (EMA), resorcinol, ammonium chloride, urea and formalin under continuous agitation in a high shear stirrer and the mixture was heated to 55 °C for 4 h. Microcapsules of different size were produced by properly adjusting the stirring rate.

### Scanning electron microscopy (SEM) and Thermogravimetric analysis (TGA)

The morphology and size distribution of the synthesized capsules was examined by SEM and the mass of the encapsulated healing agent was determined by TGA analysis as reported in our previous work Kosarli et.al^25^ and described in Supplementary Information, Section 1.3 and 1.4 respectively.

### ^1^H NMR Spectroscopy

Solid-state NMR spectroscopy was employed to investigate the effect of microcapsule size on the amount of encapsulated healing agent, which was also examined by TGA. NMR measurements were performed on all six batches corresponding to microcapsules of 6 different diameters (64–410.9 μm). To achieve proper sampling of the entire capsule production, each batch was divided in 5 distinct samples. All samples were carefully weighed to have the same mass (7 mg). Room temperature experiments were conducted in two static magnetic fields: in the 4.7 T field of a Bruker superconducting magnet having a proton resonance frequency of 200 MHz and in the 0.29 T field of a Halbach permanent magnet which is used with the portable NMR spectrometer and corresponds to a proton resonance frequency of 12.4 MHz. For the high field experiments a 200 MHz, Bruker MSL probe was used and the samples were placed in cylindrical sample holders of 5 mm diameter and 20 mm length. The experiments in the Halbach magnet were performed using a home-made coil of 10 mm diameter and 50 mm length.

All spin–spin relaxation NMR measurements were performed by applying the Carr-Purcell Meiboom-Gill (CPMG) spin echo decay (SED) pulse sequence described in detail in the Supplementary Information. The typical CPMG pulse sequence consists of a π/2 pulse followed by a train of π-pulses (π/2 – τ – (π – 2τ)_n_), which generates an equal number of consecutive spin echoes at halfway between each π-pulse pair. The spin-echo envelope was inverted by means of a Tikhonov regularization algorithm, yielding the corresponding distribution of T_2_ values as explained in the Supplementary Information. In the high field experiments (200 MHz) the π/2 pulse length was set at 1 μs and the echo decay envelope was obtained with a total of 80 pulses with an inter-pulse spacing of 500 μs. For the NMR experiments conducted in the Halbach magnet the π/2 pulse length was 5 μs. NMR experiments during thermal cycling were carried out at ^1^H Larmor frequency of 200 MHz. The thermal cycle comprised cooling the samples to − 30 °C and subsequent heating to + 60 °C in three sequentially repeating cycles starting at room temperature (+ 25 °C) and following the path: + 25 °C to − 30 °C to + 60 °C to − 30 °C to + 60 °C to − 30 °C to + 60 °C to + 25 °C. NMR measurements were obtained at room temperature and at − 30 °C and + 60 °C which are typical test temperatures in aerospace applications. The temperature was controlled by an Oxford ITC5 temperature controller with an accuracy of $\pm 0.1 \mathrm{K}$. The samples were placed in an Oxford 1200CF continuous-flow cryostat and a 4 h time window was allowed at each temperature before collecting data.

## Supplementary Information

Supplementary Information.

## Data Availability

The data that support the findings of this study are available from the corresponding author upon reasonable request.
