# Supplementary material for: Structural integrity and healing efficiency study of micro-capsule based composite materials via 1H NMR relaxometry
Source: Sci Rep. 2023 Jul 27;13:12189. doi: 10.1038/s41598-023-39302-3 (PMC10374639; doi:10.1038/s41598-023-39302-3)
Supplement: Supplementary file 1 — Supplementary Information. [file 41598_2023_39302_MOESM1_ESM.pdf]

Supplementary information:

Structural Integrity and healing efficiency study of micro-capsule based composite materials via ^{1}H NMR spectroscopy.

S. Orfanidis[1,2], M. Kosarli[2], M. Karagianni[1], M. Fardis[1], A.S. Paipetis[2], G. Papavassiliou[1]
1 Institute of Nanoscience & Nanotechnology, NCSR Demokritos, 15310 Aghia paraskevi, Attiki, Greece
2 Department of Materials Science & Engineering, University of Ioannina, Ioannina 45110, Greece

In this document, we provide details on the following topics:
1. Materials and methods.
2. ^{1}H NMR Method.
3. Tikhonov regularization algorithm.
4. ^{1}H NMR signal intensity correlation with damage rate experimental procedure.
5. ^{1}H NMR thermal cycling experimental procedure.

1. Materials and methods

1.1. Materials

Epoxy resin diglycidyl ether bisphenol-A (DGEBA, Epikote 828) supplied by Dichem Polymers, Greece was used as healing agent. Prior to encapsulation, the epoxy was diluted using ethylphenyl acetic acid (EPA at 5 % w/w), a non-toxic solvent, to reduce the viscosity of the resin. The microcapsule wall was formed using urea (NH_2CONH_2) and formalin (37 wt% in H_2O), while resorcinol (C_6H_4-1,3-$(OH)_2$) and ammonium chloride (NH_4Cl) were chosen as stabilizers. Finally, poly (ethylene-alt-maleate-anhydride) copolymer powder (EMA, Mw ¼ 100,000-500,000 g/mol) was used as a surfactant. All materials were purchased from Sigma-Aldrich and used as received. For the Tapered Double Cantilever Beam (TDCB) geometry specimens, Epikote 828 lvel epoxy resin was used as the matrix and cured with Epikure 541 (cycloaliphatic amines). Aluminum trifluoride (III) (Al (OTf)3) was chosen as the catalyst, which was supplied by Sigma-Aldrich.

1.2. Encapsulation process

The encapsulation composition was performed in our previous work Kosarli et al.[25] as will be described below.

Microcapsules were produced by in situ polymerization of oil emulsion in water. More specifically, 100 mL of deionized water was mixed overnight with 2.5 g of EMA powder in a warm bath to create a 2.5% (w/v) aqueous solution as surfactant. For encapsulation, 100 mL of deionized water was placed in a high shear stirrer Dispermat AE, VMA-GETZMANN DMBH, Germany) with 25 mL of 2.5% (w/v)

EMA solution at room temperature under continuous stirring. 0,25 g of resorcinol, 0,25 g of ammonium chloride and 2,5 g of urea were added sequentially to the solution. The pH was then adjusted from 2,7 to 3,5 by drop-wise addition of sodium hydroxide (NaOH) solution. The encapsulated agent was diluted with EPA (non-toxic solvent) at a dilution of 5 wt% to reduce the viscosity of the resin and 60 mL of this mixture was dispersed in the beaker. Six microcapsule batches were prepared at 200 rpm, 300 rpm, 400 rpm, 500rpm, 600 rpm and 800 rpm respectively, resulting in different capsule diameters. After 10 minutes, 6,33 g of formalin were added and the temperature was increased to 55°C at a rate of 10°C /min. The reaction continued at the same temperature and stirring rate for 4 h. After the bath was cooled, the microcapsules were recovered by filtration using a Buchner filter and rinsing with deionized water. The isolated microcapsules were placed in an oven at 30°C for drying for 48 h.

1.3. Scanning electron microscopy (SEM)

The microcapsules size and morphology were studied by SEM and subsequent image analysis using a JEOL JSM 6510LV scanning electron microscope, Oxford Instruments. Samples were initially coated with a gold sputter coating to prevent loading of the capsules. The selected voltage was 5 kV and the working distance (WD) was set at 15 mm. The capsule diameter was calculated from the micrographs obtained using JEOL analysis software from at least 200 measurements for each system.

1.4. Thermogravimetric analysis (TGA)

The thermogravimetric analysis (TGA) was performed in our previous work (Kosarli et al.)[25]. The main points of this analysis and most relevant to the present work are outlined in the following:

The mass of encapsulated self-healing agent (SHA) of the microcapsules was determined by TGA. TGA measurements were performed on the STA 449C Netzsch-Geratebau GmbH, Germany. Three different experimental procedures were performed. The first procedure involved a dynamic step from 25 to 600 ^{o}C at a heating rate of 10 ^{o}C /min in a flowing nitrogen (N_2) atmosphere to examine the mass loss of microcapsules in all dimensions under non-isothermal conditions. The second, involved two consecutive heating steps: a dynamic step from 25 to 180 ^{o}C at a heating rate of 10 ^{o}C /min and an isothermal step at 180 for 2 h in a flowing nitrogen (N_2) atmosphere. The third, involved two successive heating steps: a dynamic step from 25 to 210 ^{o}C, with a heating rate of 10 ^{o}C /min and an isothermal step at 210 for 2 hours, in a flowing nitrogen (N_2) atmosphere.

2. ^{1}H NMR T_2 relaxation time.

The typical CPMG pulse sequence consists of a π/2 pulse followed by a train of π-pulses (π/2 – τ – (π – 2τ)$_n$), which generates an equal number of consecutive spin echoes at halfway between each π-pulse pair. This is shown schematically in the upper panel of Figure S1. The amplitude of successive echoes decays gradually with the characteristic time constant T_2 and the entire SED curve is obtained in a single CPMG experiment from the echo envelope. The amplitude of the nth echo, formed at time $t = n \cdot (2\tau)$ is given by relation

$$M(t) = M(0) \cdot \exp\left[-\left(\frac{n \cdot 2\tau}{T_2}\right)\right] = M(0) \cdot \exp\left[-\frac{t}{T_2}\right] \quad \text{(S1)}$$

By implementing a 1-D Tikhonov regularization algorithm as described below the SED curve can be inverted to obtain the distribution $g(T_2)$ of T_2 values. The lower panel of Figure S1 illustrates the CPMG SEDs and the corresponding $g(T_2)$ distributions for the six different microcapsules batches.

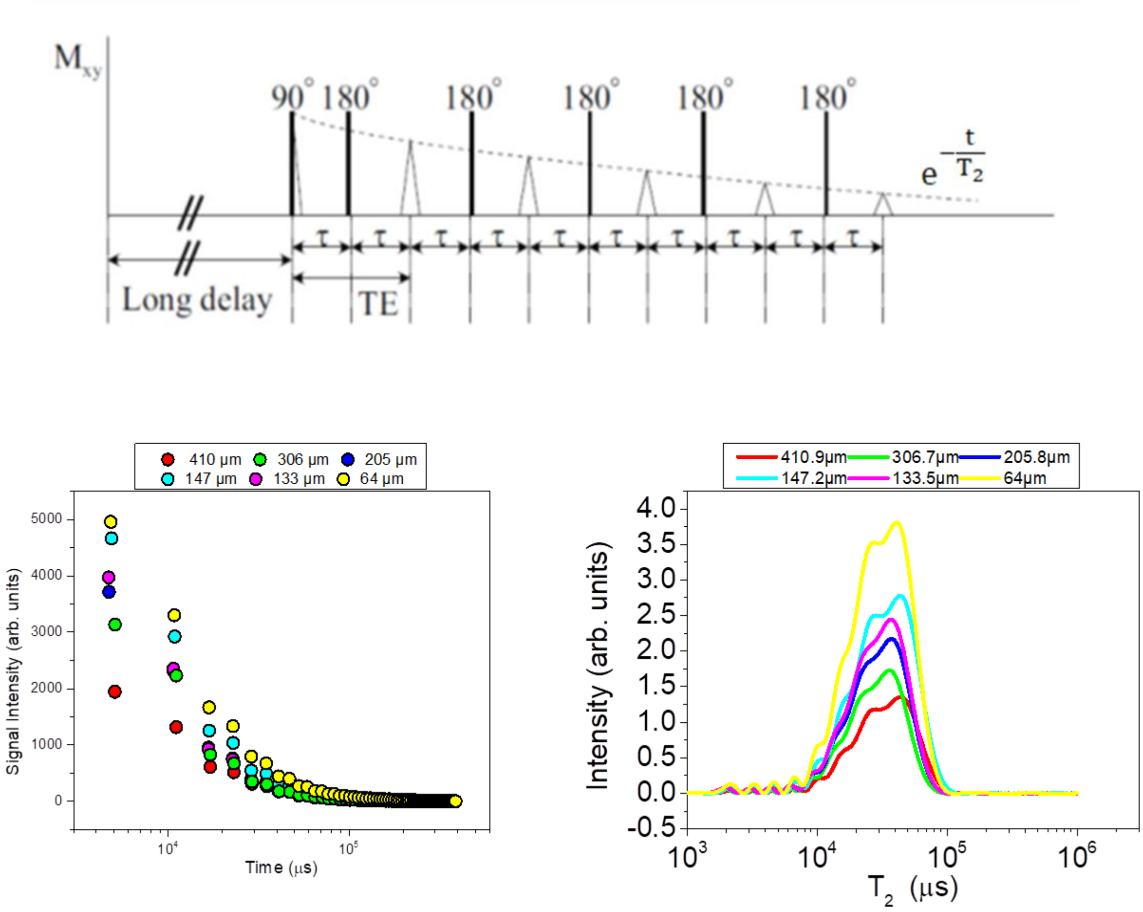

Figure S1: *upper panel*: schematic drawing of the CPMG pulse sequence. *Lower panel*: CPMG SEDs (left) and the corresponding $g(T_2)$ distributions (right) for the six different microcapsules batches.

Taking into consideration that the sample mass and the experimental acquisition parameters were kept the same for all capsules batches it is evident from Figure S1 that the signal intensity increases with decreasing capsule size.

In case of unrestricted diffusion in a constant magnetic field gradient G the amplitude of the nth echo, formed at time $t = n \cdot (2\tau)$ is given as:

$$M(\tau, G) = M(0) \cdot \exp\left[-\left(\frac{1}{T_2} + \frac{1}{3}\gamma^2 G^2 D \tau^2\right) \cdot t\right] = M(0) \cdot \exp\left(-\frac{\mathrm{t}}{T_2'}\right) \qquad \text{(S2).}$$

where γ is the nuclear gyromagnetic ratio and D the self-diffusion coefficient.
In this case, the spin echo signal intensity decreases exponentially with the effective spin-spin relaxation rate $1/T_2'$:

$$\frac{1}{T_2'} = \frac{1}{T_2} + \frac{1}{3}\gamma^2 G^2 D \tau^2 \qquad \text{(S3)}$$

encompassing both the diffusion D and the transverse relaxation T_2 .
As can be inferred from equation S3, the effect of diffusion on the CPMG SED can be made negligibly small by reducing the time spacing between consecutive π-pulses (2τ→0).

A Hahn spin echo decay of the protons in the urea-formaldehyde (UF) powder without the presence of any liquid is shown in Fig 3a. It is observed from Fig 3a that beyond a pulse spacing τ of 55 μsec the magnetization of the UF protons has completely decayed, establishing in this way a lower limit for the pulse spacings used in the employed pulse sequences.

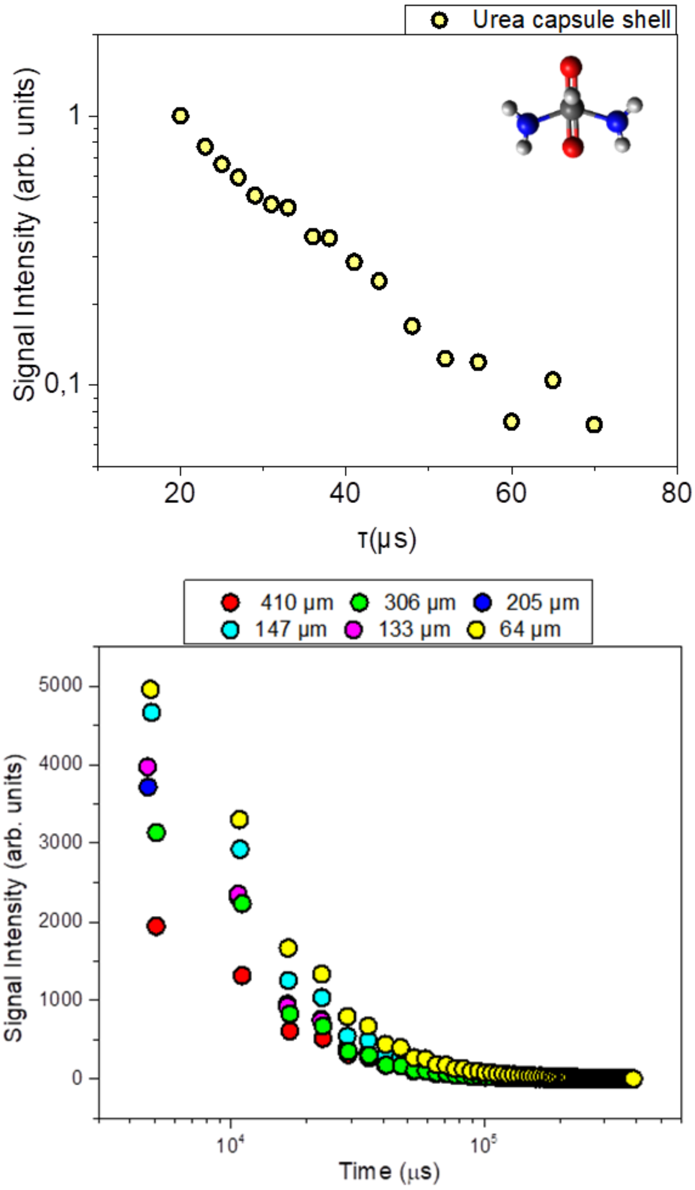

Figure S2. a) ^{1}H spin echo signal intensity as a function of the pulse spacing τ for urea-formaldehyde (UF) powder. b) Characteristic ^{1}H spin echo decays for the five different capsules batches.

3. Tikhonov regularization algorithm.

The CPMG echo envelope decay curves were inverted by implementing a 1-D Tikhonov regularization algorithm, to yield the corresponding $g(T_2)$ distributions of the spin-spin relaxation times T_2. In particular, the experimental CPMG echo amplitude envelope was modeled with a Fredholm integral equation of the first kind [1,2],

$$\frac{M(t)}{M(0)} = \int_0^{+\infty} k_0(T_2, t) g(T_2) d(log_{10} T_2) \qquad \text{(S4).}$$

In the above relation $\frac{M(t)}{M(0)}$ is the normalized signal intensity and the Kernel function $k_0(T_2, t)$ is equal to $k_0(T_2, t) = exp\left(-\frac{t}{T_2}\right)$. This equation is transformed into a discrete vector matrix formulation $M = K_0 g$, which can be inverted by implementing a non-negative Tikhonov regularization algorithm to acquire $g(T_2)$.

4. ^{1}H NMR signal intensity correlation with damage rate - experimental procedure.

In order to correlate the ^{1}H NMR signal intensity with the damage rate, an experiment was developed according to the required specifications for future use in the production line.

In particular, six different batches of microcapsules were produced, corresponding to different capsule diameters. Each batch was divided into 5 different samples that were carefully weighed to have all the same mass (7 mg) in order to achieve proper sampling of the entire capsule production. The ^{1}H NMR spin-echo decays (SEDs) acquired from all batches are illustrated in Figure S3. The variations in signal intensity between samples from the same batch are observed to increase with decreasing capsule size. Given that the decrease in observed signal intensity is attributed to the reduced mass percentage of the self-healing agent, the increased signal fluctuations observed in the batches with the smaller microcapsules indicate a higher percentage of defective capsules in these batches.

This further demonstrates the necessity of non-destructive capsule quality assessment since, as discussed in Section "*Monitoring the structural integrity of the produced capsules*" and illustrated in Figure 6 of the main article, smaller and more fragile capsules are also the most commonly used, thus increasing the risk of introducing ineffective microcapsules into the polymer matrix of the composite final structure.

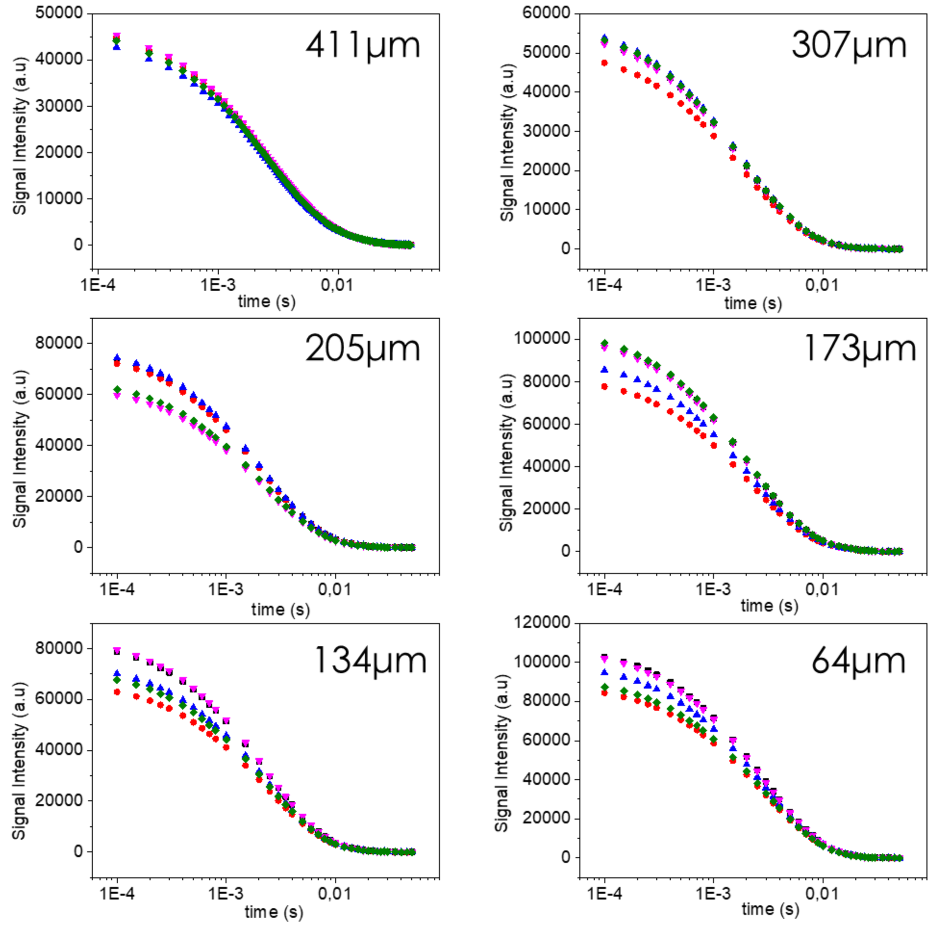

Figure S3: The NMR signal fluctuation of the six different micro-capsules batches.

5. ^{1}H NMR thermal cycling experimental procedure.

In order to simulate real operating conditions, where the materials are subjected to temperature changes from -30 ^{o}C to +60°C, thermal cycling experiments consisting of three consecutive thermal cycles were conducted.

Specifically, each cycle involved cooling the sample to -30 ^{o}C with direct exposure to liquid nitrogen flow, acquiring the ^{1}H CPMG SED after a 4-hour waiting time, and then heating the sample to +60°C, again allowing a 4-hour waiting time before acquiring the ^{1}H CPMG SED. The overall thermal path followed was:

+25 ^{o}C to -30 ^{o}C to +60 ^{o}C to -30 ^{o}C to +60 ^{o}C to -30 ^{o}C to +60 ^{o}C to +25 ^{o}C.

Experiments were performed on three microcapsule batches comprising capsules with outer diameters of 410.9 μm, 147.2 μm and 64 μm. For each batch T_2 values were also acquired at room temperature (+25 ^{o}C) before and after the thermal fatigue test.

The experimental results are presented in Figure 9 of the main article and verify that the smaller capsules are more susceptible to thermal fatigue than the larger ones, a result to be expected since the smaller capsules were shown to have the thinnest capsule shell and the highest damage rate (Fig. 6 of the main article).

References

(1). J. Mitchell, T. C. Chandrasekera, L. F. Gladden, Numerical estimation of relaxation and diffusion distributions in two dimensions. *Prog. Nucl. Magn. Reson. Spectroscopy* ***62***, 34-50 (2012).
(2). J. Day, On the inversion of diffusion NMR data: Tikhonov regularization and optimal choice of the regularization parameter. *Journal of Magnetic Resonance* ***211***, 178-185 (2011).
